# A non-invasive nanobody probe for high precision mapping of Lck spatial distribution

**DOI:** 10.3389/fimmu.2024.1440499

**Published:** 2024-07-03

**Authors:** Ioannis Tyritidis, Evangelos Tsioupros, Pantelis Christou, Nikolaos Koutras, Vasileios Morfos, Konstantina Nika

**Affiliations:** ^1^ Department of Biochemistry, School of Medicine, University of Patras, Patras, Greece; ^2^ Department of Molecular Immunology, Faculty of Biology, Institute of Biology III, University of Freiburg, Freiburg, Germany

**Keywords:** nanobody, Lck, T cells, non-invasive probe, protein localization, membrane anchors

## Abstract

The tyrosine kinase Lck is mandatory for initiating signaling responses downstream the antigenic T cell receptor (TCR). Numerous studies have shown that a prerequisite for efficient and well-balanced Lck regulation and function is its finely orchestrated spatial distribution pattern, especially at the plane of the plasma membrane. There is a wealth of knowledge on Lck localization sites, preference for specialized lipid microenvironments and colocalization partners. However, several questions concerning the spatial organization of its differentially phosphorylated conformers and the dynamics of their juxtaposition in relation to ligated and non-ligated TCRs remain elusive. In this brief report we introduce a non-invasive nanobody-based approach for mapping Lck subcellular allocation with high precision. Our initial data using this methodology, provide insight into the topology of Lck in resting T cells and its confined localization in a strictly delimited environment within the plane of the plasma membrane.

## Introduction

The Lymphocyte-specific protein tyrosine kinase (Lck), a member of the Src family kinases (SFKs) is indispensable for T cell development, proliferation and activation. The primary function of Lck is the phosphorylation of ITAM (Immunoreceptor Tyrosine-based Activation Motifs) tyrosines within the T cell receptor (TCR) complex. After TCR engagement, phosphorylated ITAMs provide anchoring sites for the zeta-chain associated protein kinase 70 (ZAP70), which is also phosphorylated and activated by Lck and which, in turn, phosphorylates additional signaling molecules mediating propagation of the signaling cascade (1). The enzymatic activity of Lck is determined by the reversible phosphorylation of two regulatory tyrosines. The conserved activating Y394, within the kinase domain, is a site of trans-autophosphorylation whereas the C-terminal inhibitory Y505 is a substrate for the C-terminal Src kinase (Csk). Both residues constitute targets of the tyrosine phosphatase CD45 (2). In resting T cells Lck exists in differentially phosphorylated states, with a significant proportion of the kinase being constitutively active (pY394-Lck), nonetheless ITAM phosphorylation is permissible only after TCR ligation. Furthermore, TCR engagement does not significantly affect the pre-existing levels of active Lck nor the equilibrium amongst the different Lck phosphospecies (3).

The simultaneous coexistence of differentially phosphorylated Lck pools and the ability of constitutively active Lck to distinguish between engaged and non-engaged TCRs point towards the notion of strictly orchestrated encounter probabilities amongst Lck, its modulators and its substrates, which are in turn regulated by tightly controlled molecular spatial patterning. Accordingly, the characterization of the lateral plasma membrane (PM) organization and spatial profile of Lck at resting and TCR-ligated T cells has been the subject of numerous studies. Lck is primarily localized at the inner leaflet of the PM, with a proportion of the protein also found in Rab11-decorated pericentrosomal compartments (4, 5). Lck membrane-anchoring is mediated by N-terminal myristoylation and di-palmitoylation, the latter being responsible for Lck partitioning into detergent-resistant membranes (6). The subcellular distribution and diffusion of Lck molecules has been shown to change in activated T cells, specifically forming clustering patterns to match those of the ligated TCRs (7, 8). However, the molecular composition of these clusters and the precise location of Lck in resting versus activated T cells still remain a conundrum (reviewed in (9).

Here we describe the development of a non-invasive and non-interfering nanobody-based approach for mapping the subcellular allocation of Lck with high precision.

Nanobodies (Nbs) are single-domain antibody fragments engineered from heavy chain-only antibodies that occur naturally in camelids; they are composed solely of one single variable heavy chain (VHH) region, which is the site of epitope binding. In recent years Nbs have been characterized as the “rising stars” of the antibody world, finding applications not only as therapeutic and diagnostic tools, but also as valuable research reagents for several methodologies including live-cell and high-resolution imaging, structural biology and proteomics (10).

To achieve selectivity for Lck, nanobodies were raised against the SH4 domain of the kinase (LckSH4), a region which shares the least sequence homology with other SFK members (11). Our methodology enables characterization of the immediate Lck localization environment and circumvents several impediments of conventional imaging and biochemical techniques.

## Results

Anti-LckSH4 Nbs were produced by the VIB Nanobody Core (Vrije Universiteit Brussel, Belgium) as previously described (12). Specifically, a Llama was immunized with an antigenic peptide corresponding to human LckSH4. Isolated Nbs were screened by biopanning and ELISA and we were provided with 30 unique cDNA sequences encoding Nbs selected for binding LckSH4 *in vitro.* Nbs are encoded by a single gene and can thus be easily expressed intracellularly by conventional gene transfer techniques. The 30 Nb-encoding cDNAs were cloned into the pLVX Tet-On lentiviral vector. This system allows inducible, reversible and tightly controlled expression of genes, where transcription is turned on by the presence of Doxycycline (Dox). Each Nb sequence contained a C-terminal HA tag, to allow detection of intracellularly expressed Nbs. The pLVX Tet-On system provided time-dependent induction of Nb expression with the highest levels being reached 48h after Dox addition (data not shown), thus all subsequent analyses were performed at this time point. Non-Dox treated cells (designated -Dox henceforth) were invariably devoid of Nb expression (as revealed by anti-HA western blots, FACS analysis and confocal microscopy) regardless of the constructs used (data not shown). Therefore, for all experimental procedures described in the manuscript, the -Dox samples are composed of a mixture of cell lines used in each corresponding experiment.

**Figure 1:**
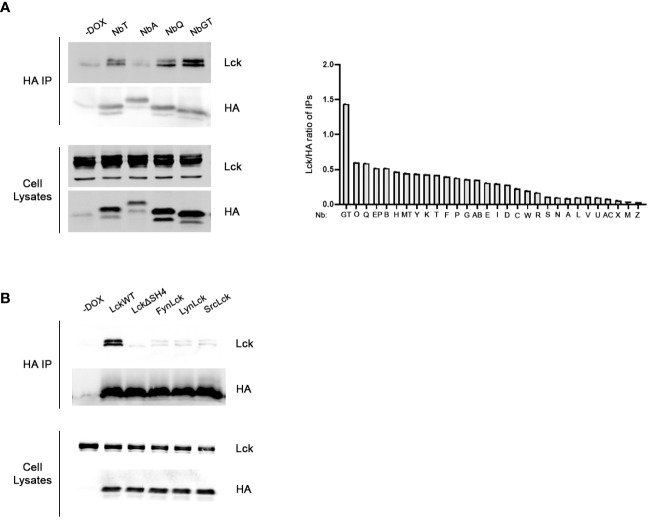
Development of Lck binding Nbs. **(A)** In-cell screening assay for the identification of the most potent binder. Constitutively expressed Lck and individual Nbs inducibly expressed under the control of Dox, were transiently cotransfected in HEK293 cells. Postnuclear lysates were subjected to immunoprecipitation using anti-HA beads and Nb-bound Lck was revealed by western blotting, as indicated. Left panel. Representative experiment screening the Lck-binding capacity of four different Nbs, as indicated. Cells cultured in the absence of Dox (-Dox sample) were used as a control for the anti-HA IPs. Corresponding blots of total cell lysates reveal expression levels of Nb and Lck constructs in the samples. The bar graph presents collective data from the screening of all 30 different anti-Lck Nbs as indicated. Immunoprecipitation potency was quantitated by densitometric analysis of the Lck bands in the immunoprecipitation blots, normalized for individual Nb expression levels as revealed by anti-HA blots of the same membranes. **(B)** Selectivity assessment of NbGT for Lck. Inducibly expressed LckWT and the indicated mutant or chimeric forms, together with NbGT were transiently cotransfected in HEK293 cells. Postnuclear lysates were subjected to immunoprecipitation as in **(A)** Corresponding blots of total cell lysates ensure equal expression of NbGT and Lck constructs in all samples.

For the identification of the strongest anti-LckSH4 Nb binder we developed a rapid and efficient in-cell screening assay. Lck and each of the 30 unique Nb sequences were transiently co-transfected into HEK293 cells. 48h after Dox addition, individual Nbs were immunoprecipitated by anti-HA beads. Samples were analyzed by Western blotting using an a-Lck antibody and coimmunoprecipitation potency was quantitated by densitometric analysis of the Lck band, normalized for individual Nb expression levels, as revealed by a-HA blots (**Figure 1A**). The in-cell screening experiments identified NbGT as the strongest binder, thus this Nb was used for all subsequent experiments.

The selectivity of NbGT against LckSH4, was evaluated by lack of co-immunoprecipation with Lck chimeric forms in which the LckSH4 domain was substituted with the corresponding domains of the SFK members Fyn, Lyn and Src or an LckΔSH4 deletion mutant (**Figure 1B**). These experiments confirmed the specificity of NbGT for Lck against other SFK members.

Having verified that NbGT was a selective and efficient detector of Lck, we used it as a probe to generate a comprehensive blueprint of Lck distribution across the plane of the plasma membrane in intact unperturbed cells. Towards this end, we reasoned that the addition of membrane anchoring motifs or transmembrane (TM) regions to NbGT’s N-terminus would enforce its partitioning within discriminate subcellular compartments and segregated spaces at the PM. By evaluating the binding capacity of N-terminally modified NbGT to Lck we would be able to profile Lck subcellular topology features.

**Figure 2:**
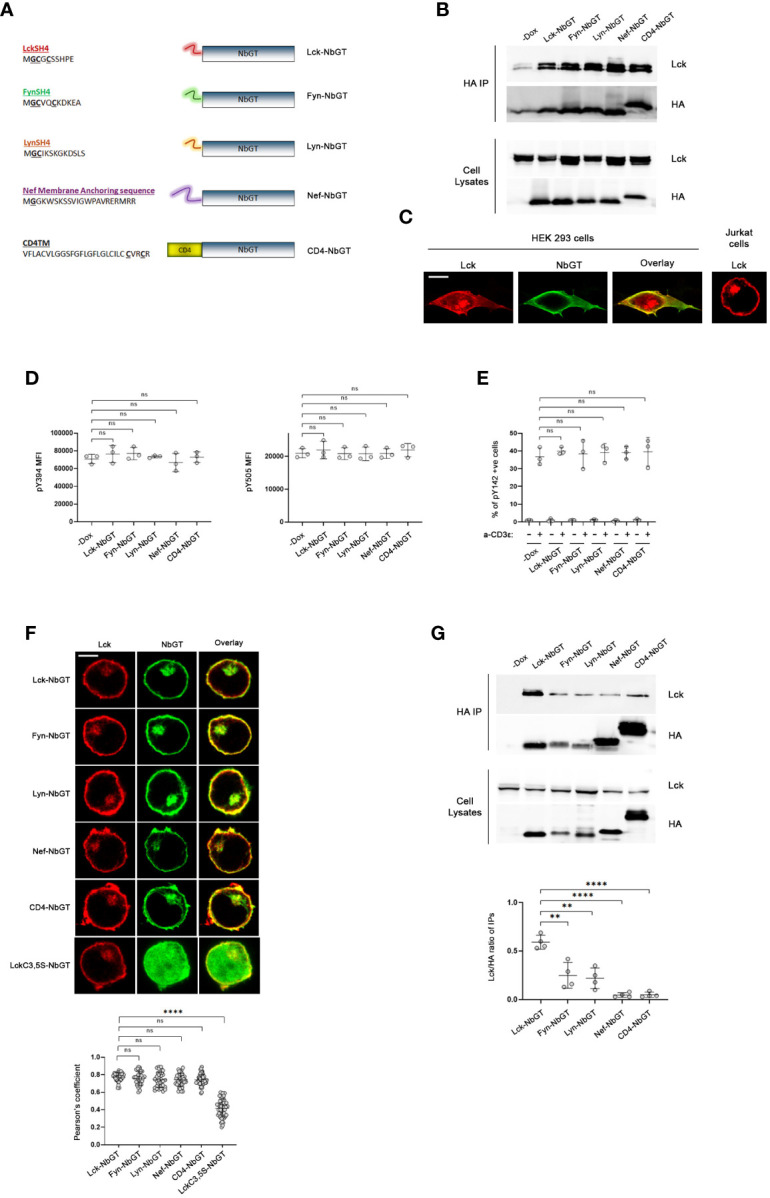
NbGT is a is a highly precise probe for Lck in intact cells. **(A)** Schematic representation of N-terminally modified NbGT constructs used in this study. Myristoylated (Glycine, G) and Palmitoylated (Cysteine, C) residues are in bold and underlined. **(B, C)** N-terminal modifications do not compromise NbGT binding to Lck. **(B)** Constitutively expressed Lck and the indicated N-terminally modified NbGT constructs were transiently cotransfected in HEK293 cells. Postnuclear lysates were subjected to immunoprecipitation as in **Figure 1A**. Western blots of total cell lysates ensure equal expression of NbGT and Lck constructs in all samples. **(C)** Representative confocal microscopy imaging of HEK293 cells co-transfected with Lck (red) and CD4-NbGT (green) as indicated. Lck staining of Jurkat cells right-hand side panel serves as a means of comparison of the subcellular distribution between endogenous (Jurkat) and exogenously overexpressed proteins (HEK 293). Scale bar, 10µm. **(D)** Expression of N-terminally modified NbGT constructs in Jurkat cells does not affect Lck regulation. Stable Jurkat cell lines expressing the indicated N-terminally modified NbGT forms and their -Dox counterparts were stained with antibodies against phosphorylated forms of the activating (Y394) and inhibitory (Y505) tyrosines and analyzed by FACS. MFI values for each antibody staining were determined by analysis with the Flowjo software. Graphs show collective data from three independent experiments (Unpaired Student t test; mean +/- standard deviation [SD]; ns: not significant). **(E)** Expression of N-terminally modified NbGT constructs in Jurkat cells does not compromise the physiological function of Lck. Stable Jurkat cell lines expressing the indicated N-terminally modified NbGT forms and their -Dox counterparts were either left untreated or were stimulated with anti-CD3ϵ for 2min at 37°C. Samples were stained with an antibody recognizing the phosphorylated form of the TCR ζ chain ITAM tyrosine 142 (pY142) and analysed by FACS. The Graph depicts frequency of cells positive for Y142 phosphorylation from three independent experiments (Unpaired Student t test; mean +/- SD; ns: not significant). **(F)** N-terminally modified NbGT constructs colocalize with endogenous Lck. Jurkat cells expressing the indicated N-terminally modified NbGT forms were immobilized on poly-D-lysine coated microscope slides, stained for Lck (red) and HA (green), and visualized by confocal microscopy. Colocalization of Lck and NbGT was quantified by the coloc2 pre-installed plugin of Image J Collective colocalization analysis results from two independent experiments are displayed on the adjacent graph. Number of cells for each sample ≥30. Scale bar, 5µm (Unpaired Student t test; mean +/- SD; ns, not significant, ****P < 0.0001). **(G)** Gradation of Lck binding amongst the different N-terminally modified NbGT constructs. Post nuclear lysates of Jurkat cells expressing the indicated N-terminally modified NbGT forms and their -Dox counterparts were subjected to immunoprecipitation using anti-HA beads and Nb-bound Lck was revealed by western blotting as in **Figure 1A**. Western blots of total cell lysates ensure equal expression of Lck and NbGT constructs in all samples. Immunoprecipitation potency was quantitated by densitometric analysis of the immunoprecipitated Lck bands, normalized for individual Nb expression levels as revealed by a-HA blots. Collective data from 4 independent experiments is depicted in the adjacent bar graph. (Unpaired Student t test; mean +/- SD; **P < 0.01, ****P < 0.0001).

Our analysis included the PM-anchoring sequences of the SFK members Lyn, Fyn and Lck itself (the latter serving as the ultimate positive control for interactions between Lck and NbGT), the helical transmembrane domain of murine CD4, extended to include the two palmitoylation sites (Cys418 and Cys422) and the membrane anchoring segment of the HIV Nef protein (residues 1-22) (**Figure 2A**). Alike Lck, attachment of Lyn and Fyn, to the inner leaflet of the PM is mediated by myristoylation and mono- or dipalmitoylation of their respective SH4 domains. Furthermore, they all share a general mechanism of regulation coordinated by the competing actions of Csk and CD45. Deductively, the membrane anchors of these SFKs should ascertain localizational similarities with Lck, which enable favorable encounters with their common regulators. Similar reasoning applies for choosing the TM region of CD4, a well-established Lck interactor (13, 14), whereas the Nef membrane anchoring sequence, notably lacking palmitoylation sites, has been shown by numerous studies to strongly colocalize with Lck both at the PM and pericentrosomal compartments (15).

A prerequisite for the validity of our studies was to verify that the addition of membrane anchors or TM regions to the N-terminal of NbGT would not compromise its epitope binding capacity. Towards this end we assessed the coimmunoprecipitation potency of N-terminally modified NbGT constructs to Lck, in HEK293 cells (**Figure 2B**). In its native T cell environment, Lck is confined at the PM with a proportion of the kinase found in pericentrosomal structures. However, when ectopically overexpressed in HEK293 cells, either due to overexpression of and/or the cellular environment, Lck displays a more dispersed and spread-out subcellular distribution (**Figure 2C**). Thus, this system provides the means to ensure protein-protein encounters that are not constrained by rules of subcellular segregation dictated by membrane anchor or transmembrane features. Indeed, all N-terminally modified NbGT constructs were capable of co-immunoprecipitating with Lck when ectopically expressed in HEK293 cells validating N-terminally modified NbGTs as suitable probes for mapping Lck cellular allocation.

We therefore proceeded to create stable Jurkat cell lines expressing all five N-terminally modified NbGT constructs under the control of Dox. To validate N-terminally modified Nbs as non-invasive probes for Lck detection we had to ensure that Lck regulation or its biological function are not affected by Nb expression. Indeed, both the phosphorylation status of Lck’s two regulatory tyrosines (pY394 or pY505) or its ability to commence TCR signaling were not compromised by Nb expression (**Figures 2D, E**), indicative of Nb binding not disturbing Lck’s physiological cellular distribution and/or diffusion pattern across the plane of the PM.

The subcellular localization of the N-terminally modified NbGT constructs was assessed by confocal microscopy. As anticipated, the incorporation of all five membrane targeting motifs provided high colocalization scores with endogenous Lck (**Figure 2F**). As a negative control for the colocalization experiments, we fused to the N-terminus of NbGT a mutant version of the Lck SH4 domain where the two palmitoylation sites (Cysteine 3 and 5) were substituted by Serines. The C3,5S SH4 mutations have been shown to alter Lck’s subcellular localization and render the kinase cytoplasmic (6). As anticipated, LckC3,5S-NbGT displayed negligible colocalization with Lck in Jurkat cells (**Figure 2F**).

Complementary to the imaging analysis, coimmunoprecipitation between Lck and individual N-terminally modified NbGTs served as a means of discrimination between protein allocation within similar subcellular compartments and adequate proximity, enabling a direct interaction. In this regard, we were pleased to observe that despite their indistinguishably high colocalization, there was definite gradation for Lck binding amongst individual N-terminally modified NbGT constructs (**Figure 2G**).

Predictably, Lck-NbGT was the most efficient in binding endogenous Lck, followed by Fyn-NbGT and Lyn-NbGT, whereas the CD4TM and Nef membrane anchor modifications prohibited coimmunoprecipitation with Lck.

## Discussion

In this study we validate an intracellularly expressed Nb, as a non-invasive probe for Lck spatial patterning in unperturbed cells. In our system, the detection reagent (NbGT) is inducibly expressed in intact cells and is allowed to bind its target prior to cell lysis or treatments with fixation and permeabilization reagents, known to frequently alter epitope targets as well as the integrity and composition of subcellular structures. We further describe an easily applied biochemical method for high-resolution protein localization that does not require alterations in target-protein composition e.g. tagging with fluorescent proteins and is not prone to inherent errors of cumbersome and complicated high-resolution imaging data analyses.

NbGT was confirmed as a valid and selective Lck intracellular detector, which did not interfere with Lck localization and biological function. Specifically, it did not cross-react with related epitopes on kinases of the Src family, it did not affect the formation of distinct Lck phosphospecies, it did not compromise the Lck-mediated triggering of TCR signaling and it accurately pinpointed the well-established sites of Lck subcellular localization i.e. the PM and pericentrosomal structures. Moreover, and importantly, in contrast to the indistinguishably high colocalization scores obtained by confocal microscopy analyses, the N-terminally modified NbGT constructs could locate native Lck with remarkable precision as revealed by gradation of coimmunoprecipitation potency.

Intuitively, the strongest association between the two proteins resulted by the incorporation of LckSH4 in NbGT’s N-terminus. Addition of Fyn and Lyn SH4 domains did not abolish, but compromised Lck association, whereas the insertion of the CD4 TM region or the membrane anchor of HIV-Nef prohibited NbGT binding to endogenous Lck in Jurkat cells.

A prerequisite for the binding of NbGT to Lck is a high probability of lateral proximity, which will permit close encounters between the two proteins. Our initial data implies an Lck residency site from which CD4 and Nef seem to be excluded and in which the SFK members Fyn and Lyn are, at least partly, present.

Furthermore, lack of CD4-NbGT binding to Lck highlights the spatial segregation of the two molecules in resting cells, despite the fact they are well-recognized binding partners; it will be interesting to determine whether this pattern is retained in TCR-ligated cells.

Our previous work (16) suggested that lateral proximity or remoteness are governed by residence of membrane anchored proteins within “like” or “unlike” boundary lipid environments, respectively. Here we provide biochemical evidence supporting this model, by showing that the strongest Lck-NbGT interaction was achieved only when the latter was directed at the PM via an identical anchoring sequence i.e. the LckSH4 domain. Interestingly, the second in ranking N-terminal modification for Lck binding was the addition of FynSH4 which presents closer resemblance to the corresponding one of Lck, translated in terms of dipalmytoylation and the presence of charged amino acids, further supporting the aforementioned model.

In conclusion, with this work we verified NbGT as a reliable non-invasive probe for Lck. Our preliminary data already provide information about the Lck residency profile in resting T cells. The incorporation of additional membrane anchoring motifs and transmembrane regions to NbGT (e.g of the TCR, CD3 and ζ chain, CD45 etc.), in combination with Lck chimeric and mutant forms, and blotting with phospho-specific Lck antibodies, will provide valuable information for the precise topology of distinct Lck phosphospecies in resting versus activated T cells as well as Lck’s biosynthetic route, both of which remain elusive.

From a wider perspective, we introduce and validate an easily applicable biochemical approach for characterizing spatial molecular segregation with high precision, and resolution superior to that of imaging techniques. Finally, by authenticating the concept of targeted Nb localization via the addition of subcellular targeting motifs, we expand the field of Nb usage as valuable probes for the non-invasive detection of molecules.

## Materials and methods

### Antibodies

HA-Tag (C29F4) Rabbit mAb (Sepharose^®^ Bead Conjugate) (Cat: 3956) used for Immunoprecipitation, Mouse anti-HA (Cat: 2367) used for western blotting, Rabbit anti-pSrc (Y416), detecting Lck pY394 (Cat: 2101), Rabbit anti-phospho-Lck (Y505) (Cat: 2751) antibodies were purchased from Cell Signaling Technology (CST). Mouse anti-Lck (3A5) (Cat: sc-433) and rat anti-CD45 (YAML 501.4) (Cat: sc-65344) were from Santa Cruz Biotechnology. Rabbit polyclonal anti-Lck (Cat: 85804) purchased from Novus Biologicals, was used for confocal microscopy. Horseradish peroxidase (HRP)-coupled secondary anti-mouse (Cat: 7076) and anti-rabbit (Cat: 7074) were from CST. For flow cytometry, mouse anti-HA tag Alexa Fluor 488 conjugate (cat: ic6875g) antibody was from R&D systems, whereas mouse anti-CD247 (pY142) Alexa Fluor 647 (Cat: 558489) antibody were from BD Biosciences. Secondary goat anti-mouse IgG Alexa Fluor 488 (Cat: A11001), goat anti-rabbit IgG Alexa Fluor 647 (Cat: A21245) and goat anti-rat IgG Alexa Fluor 568 (Cat: A11077) were from Thermo Fischer Scientific. For stimulation assays, mouse purified anti-human CD3 (clone UCHT1, Cat: 300402) antibody was purchased by BioLegend.

### Cloning and plasmids

cDNA sequences of camelid-derived nanobodies against LckSH4 with a C-terminal HA tag were produced by the VIB Nanobody Core (Vrije Universiteit Brussel, Belgium). *hLck* WT cDNA cloned in the pEF vector was used for constitutive expression of the kinase. The Lenti-X Tet-On-Advanced Inducible Expression System (Clontech Laboratories, Inc) was used for protein expression under the control of Dox. This system contains the pLVX-Tet-On-Advanced vector (constitutively expressing the tetracycline-controlled transactivator rtTA-Advanced) and the pLVX-Tight-Puro plasmid (in which the gene of interest is cloned). The lentiviral packaging plasmids pVSVG and pSPAX2 were purchased from Addgene.

N-terminally modified NbGT constructs and Lck deletion mutant and chimeras were generated by PCR using oligonucleotides comprising the corresponding membrane anchor sequences or TM regions (primer sequences are listed in **Table 1**). Nb cDNAs were cloned in pLVX-Tight-Puro, between 5’ BamHI and 3’ EcoRI restriction sites and LckWT, deletion mutant and chimeras between 5’ NotI and 3’EcoRI.

**Table 1:** List of primers used in this work.

	FW Primers	Rv Primers
N-terminally modified NbGT constructs		
Lck-NbGT	CGGGATCCATGGGCTGTGGCTGCAGCTCACACCCGGAACAGGTGCAGCTGCAGGAGTCT	CGGAATTCTCATGCGT AGTCCGGAACGTCGTA
Fyn-NbGT	CGGGATCCATGGGCTGTGTGCAATGTAAGGATAAAGAAGCACAGGTGCAGCTGCAGGAGTCT	
Lyn-NbGT	CGGGATCCATGGGATGTATAAAATCAAAAGGGAAAGACAGCTTGAGTCAGGTGCAGCTGCAGGAGTCT	
Nef-NbGT	CGGGATCCATGGGCGGCAAATGGAGCAAAAGCAGCGTGATTGGCTGGCCGGCGGTGCGTGAACGTATGCGTCGTCAGGTGCAGCTGCAGGAGTCT	
CD4-NbGT	CGGGATCCATGGAGGAGGCCGTGTTCCTGGCTTGCGTGCTGGGTGGCTCCTTCGGCTTTCTGGGTTTCCTTGGGCTCTGCATCCTCTGCTGTGTCAGGTGCCGGCAGGTGCAGCT	
LckSH4 chimeras/deletion mutant		
FynLck	ATAAGAATGCGGCCGCATGGGCTGTGTGCAATGTAAGGATAAAGAAGCAGATGACTGGATGGAAAACATC	CGGAATTCTCAAGGCTGAGGCTGGTACTG
LynLck	ATAAGAATGCGGCCGCATGGGATGTATAAAATCAAAAGGGAAAGACAGCTTGAGTGATGACTGGATGGAAAACATC	
SrcLck	ATAAGAATGCGGCCGCATGGGTAGCAACAAGAGCAAGCCCAAGGATGCCGATGACTGGATGGAAAACATC	
LckΔSH4	ATAAGAATGCGGCCGCATGGACTGGATGGAAAACATCGATGTG	

### Cell cultures and stimulation

All cell lines were kept at logarithmic growth in a humidified incubator (37°C, 5% CO2). Human embryonic kidney (HEK) 293 cells were cultured in Dulbecco’s modified Eagle’s medium (DMEM; Gibco) supplemented with 10% fetal bovine serum (FBS) (Gibco). Jurkat parental and transduced cell lines were cultured in RPMI 1640 Medium, supplemented with 10% FBS.

For Jurkat lines stimulation, cells were incubated with 1 µg/μl of soluble Anti-Human CD3ϵ (clone UCHT1) at 37°C for 2 min.

### Transient transfection

HEK293 cells were routinely transfected at 60% confluency using a Polyethylenimine (PEI, Polysciences)-based protocol. In brief, PEIpro solution was added to 200μl of Optimem (Gibco) containing the plasmids to be transfected. The mixture was immediately vortexed, incubated for 20 minutes at room temperature, and then added dropwise to the cells. After 6 hours of incubation at 37°C, the medium was replaced with fresh one and 3μg/ml of doxycycline (DOX, Sigma-Aldrich) was added to cells. The cells were grown in the presence or absence of DOX for 48 hours prior to each experiment.

### Generation of inducible Jurkat lines by lentiviral transduction

HEK 293 cells were used as a packaging line for lentivirus production. Cells were routinely transfected at 60% confluency using the PEI-based protocol described in the Transient Transfection section. The lentiviral transfer plasmids pLVX-Tet-On-Advanced and pLVX-Tight-Puro were respectively mixed with the packaging plasmids pVSVG and pSPAX2. Lentiviral particles were collected after 48 h and used for transduction. 5x10^5^ Jurkat cells were incubated with the HEK293 supernatants for 24h in the presence of 5 µg/ml hexadimethrine bromide (Polybrene, Sigma-Aldrich). After this time, HEK293 supernatants were discarded, replaced by fresh RPMI/10% FBS and cells were allowed to recover for 2 days at 37°C/5% CO2, prior to selection with 5 µg/ml Puromycin and 200 µg/ml Geneticin.

Transduced protein expression was induced by addition of by 3 µg/ml Dox to the culture medium, routinely 48h prior to each experiment.

### Coimmunoprecipitation and western blotting

Cells were lysed in ice-cold Lysis buffer (20 mM Tris-HCl pH 7.5, 150 mM NaCl, 1% NP-40, 0.5% n-Dodecyl-β-D-maltoside, 1 mM Na3VO4 and Protease Inhibitor Cocktail 5 (AppliChem)) for 15 minutes on ice and the lysates were centrifuged at 14,000g for 15 min at 4°C. The protein concentration of post nuclear lysates was determined using the Bradford protein assay reagent (AppliChem). 10 μl of anti-HA (C29F4) Rabbit mAb (Sepharose^®^ Bead Conjugate) were added to lysates containing 500μg of total protein for 2 hours at 4°C, with gentle agitation on a tube rotator. After incubation, immune complexes were washed 2 times with ice-cold lysis buffer, and then were boiled in Laemmli buffer for 10 minutes at 95°C.

Immunoprecipitates and cell lysates were separated in 12% SDS-PAGE gels and transferred to nitrocellulose membranes (GE Healthcare) using the Trans-Blot^®^ SD Semi-Dry Transfer Cell (Bio-Rad). Membranes were blocked for 1h at RT in 5% BSA diluted in TBST (0.05% Tween 20, pH 7.6) prior to incubation with primary and then HRP–conjugated secondary antibodies. Western blots were imaged using the ChemiDoc MP Imaging System (Bio-Rad).

Immunoprecipitation potency and gradation was quantified by densitometric analysis of Lck bands, normalized for Nb expression levels (HA bands) using the ImageJ software (National Institutes of Health, available at http://rsbweb.nih.gov/ij/). Statistical analysis was performed with Prism (GraphPad Software).

### Flow cytometry

Cells were fixed with BD Phosflow™ Fix Buffer I (BD Biosciences) for 10 minutes at 37°C and then incubated with permeabilization buffer (0.5% BSA, 0.5% OmniPur Triton X-100 Surfactant (Millipore)in PBS) for 20 minutes at 37°C, prior to addition of primary antibodies diluted in wash buffer (0.5% BSA, 0.1% Tween 20 in PBS), for 1 hour at 37°C. Samples were washed twice and where needed, incubated with fluorescent-coupled secondary antibodies. Samples were washed twice and analyzed on a BD Accuri™ C6 Plus Flow Cytometer. Mean fluorescence intensity (MFI) values and % of cells in the flow cytometry-analyzed samples were determined by the FlowJo Software (BD Biosciences). Statistical analysis was performed with Prism (GraphPad Software).

### Immunofluorescence staining and confocal microscopy

For confocal microscopy analysis, single-cell suspensions were immobilized on poly-D-lysine (Gibco)-coated coverslips (Marienfeld Superior) for 30 min at 37°C, fixed with BD Phosflow™ Fix Buffer I (BD Biosciences) for 10 minutes at 37°C, washed with DPBS (1X), permeabilized with permeabilization buffer (0,1% OmniPur Triton X-100 Surfactant (Millipore), 1%BSA in DPBS) for 5 minutes at 37°C and blocked with Blocking/Wash Buffer (0.1% Tween 20 (AppliChem), 1% BSA in DPBS) for 10 minutes at 37°C.

Samples were incubated with the indicated primary antibodies, diluted in Blocking/Wash Buffer for 1 h at 37°C, washed three times and incubated for 1 h at 37°C with the corresponding fluorescent-conjugated secondary antibodies, diluted in blocking/wash buffer. After three washes coverslips were mounted on microscope slides, using the ProLong Gold Antifade reagent (Thermo Fischer Scientific). Images were acquired in the Leica TCS SP5 confocal scanning microscope (Leica Microsystems) with a step size of 1.1 μm, using 488, 543 and 633 nm lasers and a 63x/1.4 oil immersion lens. Colocalization analysis of raw images was performed using the Coloc2 plugin of the ImageJ software. Regions of interest (ROI) drawn around the peripheral Lck staining were used for every cell. One single mid-section z-stack was used for each colocalization measurement.

### Statistical analysis

Statistical analysis was performed using GraphPad Prism 9 (GraphPad Software). Data were analysed using unpaired two-tailed Student t test and all data are presented as mean +/- SD. A p-value less than 0.05 was considered significant (*P < 0.05, **P < 0.01, ***P < 0.001, ****P < 0.0001; ns: not significant). All experiments were repeated with sufficient reproducibility.

## Data availability statement

The original contributions presented in the study are included in the article/supplementary material. Further inquiries can be directed to the corresponding author.

## Ethics statement

Ethical approval was not required for the studies on humans in accordance with the local legislation and institutional requirements because only commercially available established cell lines were used. Ethical approval was not required for the studies on animals in accordance with the local legislation and institutional requirements because only commercially available established cell lines were used.

## Author contributions

IT: Investigation, Methodology, Visualization, Writing – review & editing. ET: Investigation, Methodology, Visualization, Writing – review & editing. PC: Investigation, Methodology, Visualization, Writing – review & editing. NK: Investigation, Methodology, Visualization, Writing – review & editing. VM: Investigation, Methodology, Visualization, Writing – review & editing. KN: Conceptualization, Methodology, Supervision, Visualization, Writing – original draft, Writing – review & editing.
